# Integrating natural gradients, experiments, and statistical modeling in a distributed network experiment: An example from the WaRM Network

**DOI:** 10.1002/ece3.9396

**Published:** 2022-10-17

**Authors:** Case M. Prager, Aimee T. Classen, Maja K. Sundqvist, Maria Noelia Barrios‐Garcia, Erin K. Cameron, Litong Chen, Chelsea Chisholm, Thomas W. Crowther, Julie R. Deslippe, Karl Grigulis, Jin‐Sheng He, Jeremiah A. Henning, Mark Hovenden, Toke T. Thomas Høye, Xin Jing, Sandra Lavorel, Jennie R. McLaren, Daniel B. Metcalfe, Gregory S. Newman, Marie Louise Nielsen, Christian Rixen, Quentin D. Read, Kenna E. Rewcastle, Mariano Rodriguez‐Cabal, David A. Wardle, Sonja Wipf, Nathan J. Sanders

**Affiliations:** ^1^ Ecology and Evolutionary Biology Department University of Michigan Ann Arbor Michigan USA; ^2^ The Rocky Mountain Biological Laboratory Crested Butte Colorado USA; ^3^ Natural History Museum of Denmark University of Copenhagen Copenhagen Denmark; ^4^ Department of Forest Ecology and Management Swedish University of Agricultural Sciences Umeå Sweden; ^5^ CONICET, CENAC‐APN San Carlos de Bariloche Rio Negro Argentina; ^6^ Rubenstein School of Environment and Natural Resources University of Vermont Burlington Vermont USA; ^7^ Department of Environmental Science Saint Mary's University Halifax Nova Scotia Canada; ^8^ Qinghai Provincial Key Laboratory of Restoration Ecology of Cold Area and Key Laboratory of Adaptation and Evolution of Plant Biota Northwest Institute of Plateau Biology, Chinese Academy of Sciences Xining China; ^9^ Department of Environment Systems Science, Institute of Integrative Biology ETH Zürich Zürich Switzerland; ^10^ Centre for Biodiversity and Restoration Ecology, School of Biological Sciences Victoria University of Wellington Wellington New Zealand; ^11^ Laboratoire d'Ecologie Alpine Université Grenoble Alpes – CNRS – Université Savoie Mont‐Blanc Grenoble France; ^12^ Department of Ecology, College of Urban and Environmental Sciences Peking University Beijing China; ^13^ Department of Biology University of South Alabama Mobile Alabama USA; ^14^ Biological Sciences, School of Natural Sciences University of Tasmania Hobart Tasmania Australia; ^15^ Department of Ecoscience and Arctic Research Centre Aarhus University Aarhus C Denmark; ^16^ State Key Laboratory of Grassland Agro‐Ecosystems, and College of Pastoral Agriculture Science and Technology Lanzhou University Lanzhou Gansu China; ^17^ Department of Biological Sciences University of Texas at El Paso El Paso Texas USA; ^18^ Department of Ecology and Environmental Science Umeå University Umeå Sweden; ^19^ Department of Biology University of Oklahoma Norman Oklahoma USA; ^20^ Mountain Ecosystems Group WSL Institute for Snow and Avalanche Research SLF Davos Dorf Switzerland; ^21^ National Socio‐Environmental Synthesis Center Annapolis Maryland USA; ^22^ Grupo de Ecología de Invasiones, INIBIOMA, CONICET Universidad Nacional del Comahue San Carlos de Bariloche Argentina; ^23^ Asian School of the Environment Nanyang Technological University Singapore Singapore; ^24^ Department of Research and Monitoring Chastè Planta‐Wildenberg Zernez Switzerland

**Keywords:** alpine plant communities, climate change, elevational gradients, global change, mountains, warming

## Abstract

A growing body of work examines the direct and indirect effects of climate change on ecosystems, typically by using manipulative experiments at a single site or performing meta‐analyses across many independent experiments. However, results from single‐site studies tend to have limited generality. Although meta‐analytic approaches can help overcome this by exploring trends across sites, the inherent limitations in combining disparate datasets from independent approaches remain a major challenge. In this paper, we present a globally distributed experimental network that can be used to disentangle the direct and indirect effects of climate change. We discuss how natural gradients, experimental approaches, and statistical techniques can be combined to best inform predictions about responses to climate change, and we present a globally distributed experiment that utilizes natural environmental gradients to better understand long‐term community and ecosystem responses to environmental change. The warming and (species) removal in mountains (WaRM) network employs experimental warming and plant species removals at high‐ and low‐elevation sites in a factorial design to examine the combined and relative effects of climatic warming and the loss of dominant species on community structure and ecosystem function, both above‐ and belowground. The experimental design of the network allows for increasingly common statistical approaches to further elucidate the direct and indirect effects of warming. We argue that combining ecological observations and experiments along gradients is a powerful approach to make stronger predictions of how ecosystems will function in a warming world as species are lost, or gained, in local communities.

## Graphical abstract

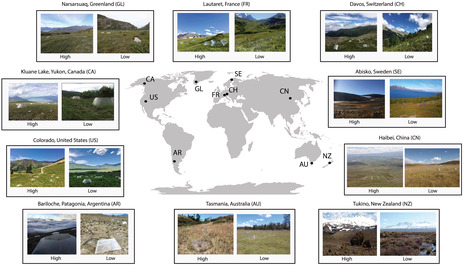

## 1 INTRODUCTION

Climatic warming impacts the functioning of ecosystems directly by affecting plant and microbial physiological processes that drive elemental cycling and indirectly by altering the phenotypes and performance of species, which in turn affects the composition and relative abundance of species, and their associated traits, within communities. Designing and implementing experiments that allow us to test, understand, and predict the impacts of both the direct and indirect effects of warming on community and ecosystem properties and processes across space and time is critical. There are now numerous experiments that manipulate air or soil temperature and measure associated community‐ and ecosystem‐level responses (Song et al., 2019; van Wijk et al., 2003), each providing mechanistic insights into the ecological responses to temperature in different locations. Because such warming experiments are often costly, they are typically conducted at single sites (Borer et al., 2014; Henry & Molau, 1997), which potentially hampers our ability to make generalizable predictions about the impact of warming. Ideally, experiments would be replicated at multiple sites across multiple climatic or temperature regimes to gain a better understanding of the impacts of warming at a global scale (Elmendorf et al., 2015; Song et al., 2019). In addition, multiple‐site comparisons are usually only explored via meta‐analyses, which can be limited by a lack of statistical power and high Type II Error resulting from combining studies with very different methodologies and measurements, approaches to the problem, and means of experimental warming.

Climate change is not the only driver of changes in community‐level traits, community structure, and ecosystem function. Dominant species, typically defined as those that are abundant and have large impacts on community dynamics and ecosystem function, are also the subject of much experimental work (Avolio et al., 2019). Changes in the abundance or identity of dominant species can have cascading impacts on species, community dynamics, and ecosystem processes (Díaz et al., 2007; Sasaki & Lauenroth, 2011). For example, in some grassland ecosystems, dominant grasses offset the negative effects of species loss by promoting overall ecosystem productivity, providing short‐term resistance to declines in ecosystem function associated with nonrandom species loss (Grime, 1998; Smith & Knapp, 2003). In other systems, dominant species can suppress the biomass of the subdominant plant community and influence overall community composition (Hillebrand et al., 2008).

Increases in both air and soil temperature directly impact the physiology of individual organisms, such as photosynthetic rates (e.g., (Reich et al., 2018) and microbial metabolic activity (Bai et al., 2013; Cavicchioli et al., 2019)), shaping how species interact with one another, ultimately scaling to influence important ecosystem functions, such as carbon and nutrient cycling and storage. Warming‐induced changes in community composition are often associated with shifts in species‐specific functional traits (Bjorkman et al., 2018), which can have cascading consequences for ecosystem carbon and nutrient dynamics (Liu et al., 2018). The relative importance of these drivers—the loss of dominant species and climate—and how they modify interactions and shape community composition may change across space, from field site to field site, and through time. Therefore, developing frameworks that enable ecologists to explore long‐ and short‐term responses to warming across ecosystems is critical.

Previous studies have shown that shifts in the functional traits of communities, especially plants, can lead to dramatic alterations in the dynamics and functioning of ecosystems (Bello et al., 2010; Diaz et al., 2004; Lavorel & Garnier, 2002; Lavorel & Grigulis, 2012). Therefore, the indirect impacts of warming on ecosystem functions (e.g., C dynamics) can be greater than the direct effects on the performance of individual organisms (Niu et al., 2011; Wipf & Rixen, 2010). The effect of shifts in plant community composition on ecosystem properties and processes can be especially pronounced if the loss of dominant species, and their associated traits, occurs (Avolio et al., 2019; Grime, 1998; Smith & Knapp, 2003). While not always the case (Díaz et al., 2007; Isbell et al., 2017; McLaren & Turkington, 2010), dominant species tend to have large and cascading influences on communities and ecosystems, often proportional to the large fraction of community biomass they make up (Avolio et al., 2019). For this reason, dominant species identity, as well as the evenness of plant communities, can be important predictors of gross respiration and photosynthesis in plant communities (Heskel et al., 2013; Orwin et al., 2014). Understanding, testing, and modeling the interactive influence of the separate and interactive effects of climatic change and shifts in plant community and trait composition on ecosystem function is critical to predicting the impact of climate change on communities and ecosystems.

Despite the multitude of warming experiments and dozens of dominant species removal experiments conducted to date (Avolio et al., 2019; Song et al., 2019), there are still significant challenges in understanding and predicting how background climate may mediate the influence of warming and species losses on the functioning of ecosystems in different environmental contexts. Put another way, is the impact of a 2°C increase in temperature the same in a cold, dry ecosystem as in a warmer and wetter ecosystem? Do dominant species exert more influence in benign environments than in stressful environments? One way to test this would be to conduct experiments along climatic gradients or in sites where climate at least differs systematically (Fukami & Wardle, 2005). This could be achieved by setting up experiments that combine warming with the removal of dominant species along gradients, or at sites representative of the climatic end members of a given gradient (i.e., high‐ and low‐elevation sites), especially in multiple regions around the world. Such a design will not only enhance our understanding of the influences of the individual and interactive effects of climatic change and associated changes in community and trait composition on ecosystem functioning but will also allow us to better predict how and why these effects may be shaped by multiple unique climate combinations and to investigate mechanisms of global relevance regardless of biogeographic history or phylogenetic or environmental contexts or legacies. However, studies exploring the consequences of warming‐associated shifts in species interactions across multiple sites are rare (but see Song et al., 2019).

While manipulating the direct and indirect effects of climate change at a global scale in a single project is challenging, we outline a new network of experiments and observations that, together with the use of causal models, will foster a comprehensive and predictive understanding of the impacts of warming on communities and ecosystem function, and how these effects differ among contrasting locations. Here, we first highlight the power of harnessing natural, systematic variation in temperature by working along elevational gradients in mountain systems. Next, we review some of the strengths and weaknesses of experimental approaches used to understand the impacts of climatic warming. We then discuss emerging statistical approaches that help us explore causal networks of direct and indirect effects of experimental manipulations such as warming and dominant species removal on communities and ecosystems. Finally, we demonstrate this approach by way of example, introducing a globally distributed network of experiments explicitly designed to test the direct and indirect effects of warming and species removal on community structure and ecosystem function across contrasting mountain systems.

**FIGURE 1:**
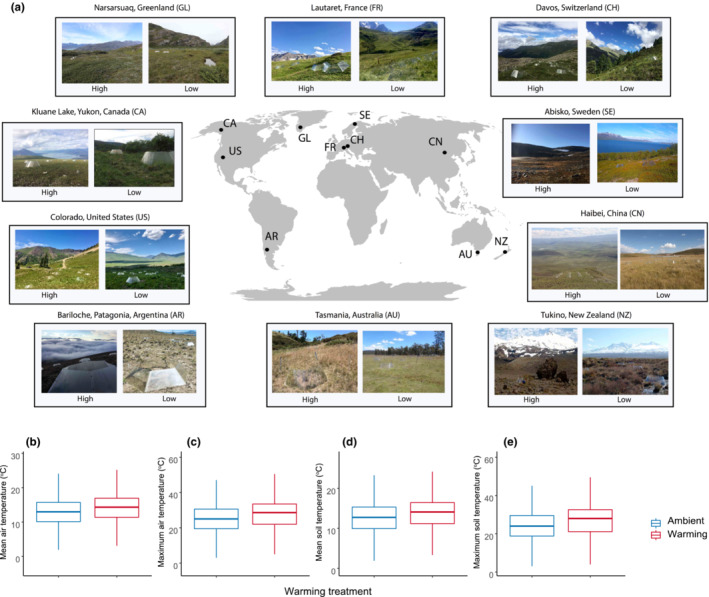
Global distribution of the 10 WaRM network locations and the effects (averaged across all 10 locations) of open‐top warming chambers on mean growing season air temperature (b) maximum growing season air temperature, (c) mean growing season soil temperature and (d) maximum growing season soil temperature; all showing increases of roughly 2°C.

The warming and species removal in mountains (WaRM) network is a novel approach that (1) uses low‐ and high‐elevation sites that differ in temperature by approximately 2°C, (2) establishes dominant species removals and passive warming chambers (increasing air temperature ~2°C) to simulate short‐term warming and shifts in species interactions, and (3) crosses experimental warming and species removals to explore the interactive effects of these treatments. This distributed experiment in 10 mountain ecosystems around the world (see Figure 1) will enable us to explore interactions among drivers and response variables in a way that will help us better understand and predict the direct and indirect effects of global warming on contrasting mountain ecosystems.

## 2 HARNESSING NATURAL ENVIRONMENTAL GRADIENTS TO BETTER UNDERSTAND THE IMPACTS OF ENVIRONMENTAL CHANGE ON PLANT COMMUNITIES AND ECOSYSTEM FUNCTION

For over 160 years, studies in ecology and evolution have employed environmental gradients to help understand how natural communities respond to macroclimate (e.g., von Humboldt 1849). Elevational gradients capture variation in temperature, soil age and type, disturbance regimes, and land‐use histories and have yielded important insights into how organisms, communities, and ecosystems vary with climatic and other abiotic conditions (Martinez‐Almoyna et al., 2019; Mayor et al., 2017; Rogora et al., 2018). Comparisons along elevational gradients, and between two points across elevational gradients, can also be used to explore the impact of temperature on the properties of species and communities and the functioning of ecosystems (Kivlin et al., 2014; Read et al., 2014) provided that the environmental factors, such as precipitation and aridity, that co‐vary with elevation (Körner, 2007) are accounted for. For example, Mayor et al. (2017) showed clear shifts in leaf nitrogen to phosphorus ratios with declining temperature along elevational gradients around the world. Mayor et al. (2017) also showed that the indirect effects of elevation‐associated changes in temperature, mediated via plant nutrient responses, were associated with changes in belowground abiotic and biotic properties across regions. In a classic study Illustrating how species interactions may vary with elevation, Callaway et al. (2002) conducted a removal experiment at 10 mountain sites around the world to demonstrate that positive interactions among species are more common in stressful, high‐elevation sites, but that competitive interactions are more common at less stressful, low‐elevation sites. An ability to understand, and contrast, ecosystem responses at high‐ and low‐elevation sites, can lead to key insights at both local (Sundqvist et al., 2020) and global (e.g., Mayor et al., 2017) scales. Thus, elevational gradients, which allow us to capture environmental heterogeneity, serve as powerful study systems for understanding both longer‐term, as well as larger‐scale, community and ecosystem responses to environmental change (Fukami & Wardle, 2005; Walker et al., 2010).

While observational gradient studies are powerful tools in ecology, they make it difficult to disentangle or isolate the role of specific abiotic and biotic factors in driving observed patterns. Moreover, many environmental factors such as temperature, moisture, soil age, and soil depth may vary with elevation, often not in synchrony (Körner, 2007) or concurrently across elevational gradients. This lack of uniformity in environmental conditions along gradients may lead to contrasting findings among studies and may complicate meta‐analyses that do not consider how such factors vary idiosyncratically with elevation. For example, studies exploring how temperature drives soil microbial biomass and community structure, or the temperature sensitivity of soil processes along elevational gradients, have yielded variable results (Carey et al., 2016; He et al., 2020), which limits our understanding of what drivers affect soil carbon stocks across contrasting ecosystems. In water‐limited ecosystems, for example, precipitation rather than temperature may exert the strongest influence on community and ecosystem properties and processes along environmental gradients (Bradford et al., 2017; McCain, 2007; Sundqvist et al., 2013). In some instances, no discernible elevation‐linked drivers of ecosystem functioning or community composition are found among elevational gradients at a global scale (Hendershot et al., 2017). One way to harness the power of environmental gradients, and to overcome some of their limitations, is to use experimental infrastructure in combination with the gradient approach (Fukami & Wardle, 2005). Combining observational work that captures environmental heterogeneity (natural experiments) with manipulative experiments, and “mega‐analysis” (i.e., analysis of the same experiment across sites, see [Kleyer et al., 2012]) enables researchers to harness the power of each of these individual approaches to better understand the impacts of warming on systems across space and through time (Borer et al., 2014; Elmendorf et al., 2015; Elmendorf, Henry, Hollister, Bjork, Bjorkman, et al., 2012).

## 3 MANIPULATIVE CLIMATE CHANGE EXPERIMENTS AND THE EFFECTS OF ENVIRONMENTAL HETEROGENEITY AND CHANGE ON COMMUNITY AND ECOSYSTEM PROPERTIES AND PROCESSES

Experiments that manipulate abiotic conditions or reciprocally transplant individuals (e.g., Alexander et al., 2016; Nooten et al., 2017) are powerful tools that can be used to understand and predict the effects of environmental change on biological communities and ecosystems. Over the past few decades, the application of manipulative global change experiments has grown exponentially (Borer et al., 2014; Song et al., 2019). However, using experiments alone to understand community‐ and ecosystem‐level responses to warming may underestimate the effects of warming, as manipulative experiments are often executed at small spatial scales, and across relatively short time scales, which cannot accurately reflect the accumulated changes that are expected from warming at the decadal scale (Kröel‐Dulay et al., 2022; Wolkovich et al., 2012). For instance, a recent study found that natural rainfall seasonality constrained the response of biomass production to CO_2_ fertilization in temperate grasslands worldwide, suggesting that the positive response of biomass production to rising atmospheric CO_2_ concentrations may be substantially less than originally predicted (Hovenden et al., 2019). Thus, there is a need for manipulative experiments that span larger abiotic gradients to understand the effects of climatic change on community and ecosystem properties and processes.

Increasing surface air and soil temperatures directly impact plant physiology and community dynamics as well as the soil microbial community structure and functioning (which is an important mediator of climate), though the direction and/or magnitude of the effect has been shown to vary considerably across ecosystems and spatiotemporal scales (Bradford et al., 2017; Carey et al., 2016; Crowther et al., 2016; Elmendorf, Henry, Hollister, Björk, Bjorkman, et al., 2012; Liu et al., 2018; Zavaleta et al., 2003). Generally, experimental warming leads to reductions in plant diversity and sometimes idiosyncratic changes in plant community composition (Cowles et al., 2016; Wu et al., 2011). However, some experimental work has found no effect of warming on plant communities or effects that are mediated by other abiotic or biotic factors (Báez et al., 2013; Zavaleta et al., 2003). Similarly, warming stimulates soil respiration at some sites, but several studies have shown neutral or negative responses to warming, thought to be attributable to moisture limitation (Suseela et al., 2012), changes in microbial community composition and functioning (Castro et al., 2010; Zumsteg et al., 2013), or acclimatization (Luo et al., 2001; Melillo et al., 2017). A better understanding of the interaction between warming, abiotic conditions, and community and ecosystem properties, as well as the context‐dependency of these factors and interactions, is necessary for us to model, predict, and adapt to global change.

In addition to manipulating abiotic conditions, explicitly testing the effects of shifts in species dominance and relative abundance with warming is critical to our overall understanding of both the direct and indirect effects of global change on biological communities and ecosystem functioning (Alexander et al., 2016). Though nondominant species can exert important effects on ecosystem functioning (Isbell et al., 2017; Jain et al., 2014; Peltzer et al., 2009), theory and experimental evidence suggest that dominant plant species typically play the largest role in shaping community composition and ecosystem dynamics. For example, in grasslands, dominant species often drive productivity (Orwin et al., 2014; Smith & Knapp, 2003). Furthermore, responses of ecosystem processes to experimental removal of dominant plant species and plant functional groups aboveground can be mediated by factors such as soil fertility and plant productivity (Fanin et al., 2018, 2019; Kardol et al., 2018) both of which can decline with elevation (Bryant et al., 2008). Collectively, these studies suggest that the responses of dominant plant species to warming, and their concomitant effects on ecosystem function, may vary across wider temperature gradients, which are often absent from site‐level manipulative climate change experiments.

## 4 THE WARM NETWORK: A TEST CASE FOR INTEGRATING GLOBAL CHANGE EXPERIMENTS AND NATURAL ENVIRONMENTAL GRADIENTS

### 4.1 Study system

**FIGURE 2:**
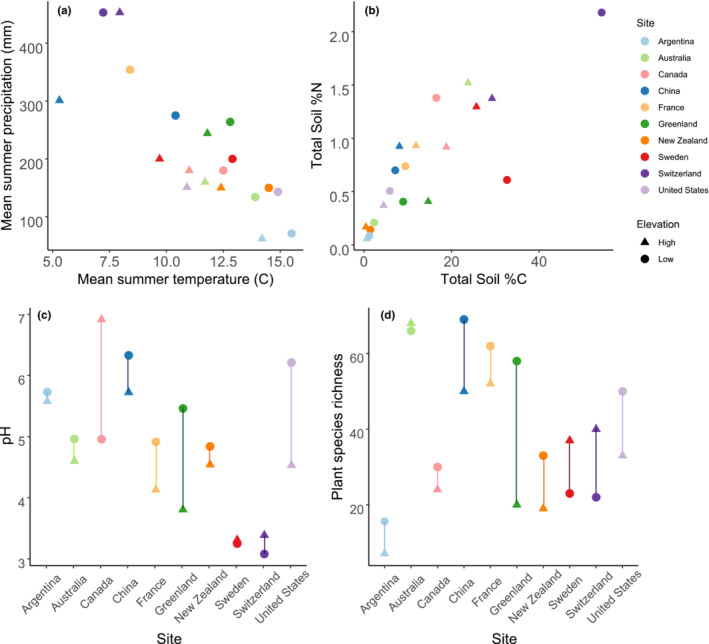
Abiotic and biotic variation among the ten sites in the WaRM network, at the high‐ and low‐elevation sites. (a) Mean summer precipitation and temperature (warmest quarter for a given site), (b) Soil C and N, (c) pH, and (d) site‐level plant species richness.

The WaRM network consists of 20 study sites distributed in ten mountain locations over five continents (North America, South America, Europe, and Austral‐Asia) ranging in latitude from 39° S to 68° N (Figure 1a; Table 1). Each of the ten locations has a high‐ and a low‐elevation site, where the difference in elevation between the two sites ranges from 252 m to 804 m (Table 1), with an average of 514 m between high‐ and low‐elevation sites across the network. During the growing season, the warmest study location is in Patagonia, Argentina, where mean summertime temperature is 15.5 and 14.2°C at the low‐ and high‐elevation sites, respectively (Figure 2a; Table 1). The coldest study site (both growing season and wintertime temperatures) is the high site in Haibei, China, where mean summertime temperature is 5.3°C (Figure 2a and Figure S1; Table 1). Patagonia, Argentina, is the driest study location with a mean growing season precipitation of 66.5 mm at the low‐elevation site, while the wettest study location is Davos, Switzerland, where mean growing season precipitation is 453 mm (Table 1). Study locations varied by both summer and winter temperature and precipitation patterns, defined by warmest and coldest quarter temperatures and precipitation values (Figure 2a and Figure S1). While some sites receive fairly consistent precipitation across the year (e.g., New Zealand), others rely more heavily on summertime precipitation (e.g., China) or wintertime precipitation (e.g., Argentina) (Figure 2a and Figure S1). For wintertime precipitation and temperature, we relied on the WorldClim database in the absence of site‐level data, which does not allow us to differentiate between elevations for five of our study sites, though we would expect slightly colder temperatures at the high‐elevation sites with more nuanced shifts in precipitation. We selected each of the 20 elevation sites so that they were devoid of trees, hence in full sunlight. We also selected the high‐ and low‐elevation sites within each study location in such a way as to minimize between‐site differences in aspect, slope, geology, plant growth form, and hydrology, in order to best isolate the impact of climate between elevations.

**TABLE 1:** List of the location of the ten study locations within the Warming and species Removal in Mountains (WaRM) network, their elevation, local climate (Hijmans et al., 2012 and plot‐level sensors; mean summertime (growing season) temperature (MST) and mean summertime (growing season) precipitation (MSP)) and soil properties (pH and C:N), and dominant vascular species at each site. At each high‐ and low‐elevation site within each study location, experimental warming by open‐top chambers (see Figure 1) is crossed with a removal of the dominant species listed at each site.

Country	Study location	Year of establishment	Elevation (m a.s.l.)	Latitude	Longitude	MST (°C)	MSP (mm)	Soil pH	Soil C:N	Dominant vascular plant species (removed)	Growth form
Sweden	Abisko	2014	894	68.294	19.099	9.7	300	3.31	19.17	*Empetrum hermaphroditum*	Woody evergreen
			498	68.314	19.163	12.9	300	3.25	48.82	*Empetrum hermaphroditum*	Woody evergreen
Greenland	Narsarsuaq	2015	450	61.155	−45.379	11.8	244	3.80	40.47	*Betula glandulosa*	Woody deciduous
			50	61.183	−45.370	12.8	264	5.46	21.72	*Betula glandulosa*	Woody deciduous
Canada	Kluane Lake, Yukon	2015	1900	60.954	−138.423	11.0	186	6.91	20.83	*Carex consimilis*	Sedge
			1431	60.979	−138.408	12.5	186	4.96	12.46	*Salix reticulata*	Woody
France	Lautaret	2017	2460	45.054	6.401	8.4	354	4.13	12.78	*Trifolium alpinum*	Forb
			1900	45.040	6.419	8.4	354	4.91	12.84	*Patzkea paniculata*	Grass
Switzerland	Davos	2014	2353	46.774	9.857	7.95	453	3.39	21.41	*Vaccinium uliginosum*	Woody deciduous
			2101	46.775	9.863	7.22	453	3.08	25.85	*Vaccinium uliginosum*	Woody deciduous
USA	Colorado	2013	3460	38.992	−107.067	10.9	151	4.53	12.23	*Juncus drummondii*	
			2740	38.715	−106.823	14.9	143	6.21	11.81	*Wyethia amplexicaulis*	
China	Haibei	2014	4004	37.707	101.372	5.3	301	5.72	8.80	*Kobresia pygmaea*	
			3200	37.617	101.2	10.4	275	6.33	10.55	*Stipa aliena*	
Australia	Tasmania	2015	890	−42.090	147.088	11.7	160	4.60	15.80	*Poa gunni*	C3 grass
			440	−42.343	147.341	13.9	134	4.96	15.17	*Austrostipa sp*.	C3 grass
Argentina	Bariloche, Patagonia	2016	1321	−41.654	−71.073	14.2	62	5.58	6.29	*Acaena splendens*	Woody
			772	−40.998	−71.088	15.5	71	5.73	4.14	*Papostipa speciosa*	Grass
New Zealand	Mt. Ruapehu, Tukino	2015	1611	−39.278	175.626	12.4	150	4.54	2.48	*Gaultheria collensoi*	Woody
			1071	−39.294	175.726	14.5	150	4.84	10.98	*Calluna vulgaris*	Woody

### 4.2 Experimental design

From 2013 to 2017, we established experimental plots at a high and a low site in each of the ten study locations (Figure 1a; Table 1) to take advantage of long‐term climatic and other differences between low (typically warmer) and high (typically colder) elevation sites. In 2013, we established a site in the *United States of America* in the Colorado Rockies. In 2014, we established sites at four locations—in *Australia* in Tasmania*, China* on the Tibetan Plateau in Haibei, *Sweden* at Abisko, and *Switzerland* at Davos. In 2015, we added sites in *Argentina* near Bariloche in Argentinian Patagonia*, Canada* in Kluane Lake in the Yukon Territory*, Greenland* at Narsarsuaq, and *New Zealand* near Mt. Ruapehu, Tukino. In 2017, we added a final site in Lautaret, *France* (Figure 1). During the first year at each of the sites, a 2 × 2 factorial warming × plant species removal experiment at both low and high elevations was installed. We established a total of 32, 1.5 m diameter plots centered on an area of 2 m × 2 m at each elevation site, resulting in a total of 64 plots at each location (except for at Abisko, Sweden, where a total of 40 plots were installed; 20 at each elevation site). Prior to treatment application, we conducted visual estimation of percent cover of all species in each plot. This visual estimation of plant species cover was followed by randomly assigning plots at each of the elevation sites within each location to one of the four treatments (in a 2 × 2 factorial design: Control (not warmed, dominant species not removed), Removal (not warmed, dominant species removed), Warming (warmed, dominant species present), and Warming × Removal (warmed, dominant species removed; *n* = 8 of each at all elevational sites, except for at Abisko, Sweden where *n* = 5)).

To experimentally raise temperature in plots assigned with a warming treatment, we used transparent hexagonal open‐top chambers (OTCs) with an inside diameter of 1.5 m and a height of ~65 cm. Open‐top chambers are commonly used in climate change experiments to raise temperature in remote areas (Elmendorf, Henry, Hollister, Bjork, Boulanger‐Lapointe, et al., 2012). We used iButtons (Thermochron & Hygrochron ibuttons, Maxim Integrated Corp., San Jose, CA, USA) placed in the center of each plot, to continuously measure air and soil temperature, as well as (in some plots) air humidity at 5 cm aboveground, and belowground, in each plot over each growing season. Across all sites, the OTCs raised the growing season mean air temperature by ~2°C and soil temperature by ~1°C (Figure 1b–e). Mean (and max) air temperature across all sites was 14.0°C (28.1°C) in warmed plots versus 12.9°C (25.0°C) in control plots. Mean (and max) soil temperature in warmed plots was 11.9°C (16.3°C) and was 11.0°C (15.3°C) in control plots. At each site, we defined dominant species as those that made up most of the total percent plant cover at that site. The identity of the dominant species often varied between high‐ and low‐elevation sites within a region and included grasses, dicots, or shrubs across a variety of families; this variation in the identity (and functional group and phylogenetic context) lets us explore whether dominant species, regardless of identity, have comparable effects across disparate regions. However, the Greenland, Sweden, and Switzerland sites removed the same species at high and low elevations (Table 1). Regardless of its taxonomic identity, we were interested in the functional effect of the dominant species at each site because dominant species most commonly have the strongest effects on community and ecosystem dynamics (Avolio et al., 2019; Grime, 1977). Dominant species removal was conducted by hand‐clipping to ground level, and clipping was maintained as necessary throughout the duration of the experiment, with all removed biomass then being dried and weighed.

### 4.3 Baseline data

Prior to the establishment of the experiments at each site, we collected baseline data including soil total carbon, total nitrogen and pH in addition to plant diversity measured by visual estimation of percent cover as described above (Figure 2a–e). Soil samples (*n* = 5 per elevational site per location) for initial site characterization were taken to a depth of 5 cm with a soil corer of 5–10 cm in diameter to minimize compaction. The specific corer dimensions as well as the number of composited cores and the total depth of each sample varied by location, and the volumes of soil collected were recorded and used to calculate accurate bulk densities. Each volumetric sample was air‐dried, and soil pH was analyzed following a protocol developed by (Minasny et al., 2011). Total soil C and N were analyzed on subsamples of the same soil samples via combustion (Leco CN628). Soil pH varied by country—and within some countries between high‐elevation and low‐elevation sites—ranging from roughly 3.5 in Switzerland to roughly 7 in Canada. We used visually estimates of plant community composition and plant species richness, and we found that plant species richness differed by site (Figure 2d), ranging from 7 and 16 total species at the low‐ and high‐elevation Argentinian sites, respectively, to 66 and 68 total species at the low‐ and high‐elevation Australian sites, respectively (Figure 2d).

We measured air and soil temperature at the plot level for most study locations and coupled those data with precipitation data derived from WorldClim (along with WorldClim‐derived temperature data for Australia, China, and France where plot‐level data were not available). Sites varied in abiotic conditions (Figure 2a–c); some sites would appear to be more temperature‐limited (e.g., China, France, and Switzerland) and some more water‐limited (e.g., Argentina, Australia, and the United States).

Following experimental establishment, we collected data designed to ask and answer questions, and test hypotheses, about the role of warming and dominant plant species, and their interaction (see Table 2), on plant and soil communities and ecosystem functioning across contrasting elevational sites around the world. The core data we collected are as follows: soil moisture, soil respiration, pH, total soil carbon and nitrogen, net ecosystem exchange, ecosystem respiration, water use efficiency, gross primary productivity, plant community composition, a suite of plant functional traits, and the normalized difference vegetation index (NDVI) derived from reflectance data as an indicator of aboveground biomass or “greenness” (Rouse et al., 1974). We laid out three overarching questions: (1) How does warming, the loss of dominant species, and the interaction between those two factors impact biodiversity, species interactions, phenology, and the functioning of montane ecosystems (e.g., the pools and fluxes of carbon and nitrogen)? (2) How does background climatic variation influence the impacts of warming and the loss of dominant species on communities and ecosystems? And (3) Are the impacts of warming and the loss of dominant species context‐dependent or are there generalizable patterns (e.g., is the impact of a 2°C increase in temperature the same in a cold, dry ecosystem as in a warmer and wetter ecosystem)?

**TABLE 2:** Selection of the foundational questions that the WaRM network is designed to ask and explore

Key Questions
1. How does warming, the loss of dominant species, and the interaction between those two factors impact biodiversity, species interactions, phenology, and the functioning of montane ecosystems (e.g., the pools and fluxes of carbon and nitrogen)?
2. How does background climatic variation influence the impacts of warming and the loss of dominant species on communities and ecosystems?
3. Are the impacts of warming and the loss of dominant species context‐dependent or are there generalizable patterns (e.g., is the impact of a 2°C increase in temperature the same in a cold, dry ecosystem as in a warmer and wetter ecosystem)?

### 4.4 Analysis potential

**FIGURE 3:**
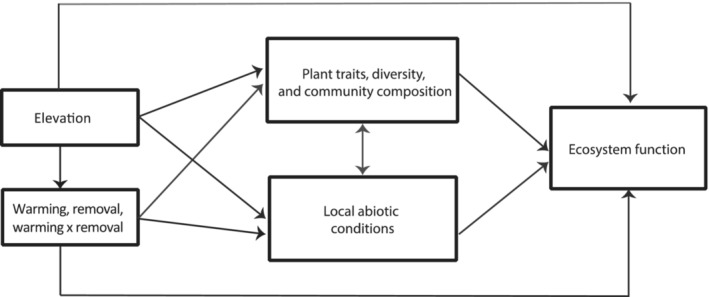
Proposed direct and indirect effects of warming on plant communities and ecosystem functioning in mountains around the world, highlighting the hypothesized relationships between these various factors and the value of uniting statistical modeling tools, like SEM, with a replicated global change experiment and observation gradients.

The WaRM network serves as a replicated, distributed global change experiment that combines the manipulation of temperature and shifts in species dominance with elevational gradients (high and low sites) at ten locations across the globe. Observational gradients and experimental techniques are useful tools for measuring and predicting the consequences of global climate change, particularly when used in combination, but statistical techniques enable us to explore interactions and indirect effects in ways that help us better understand the complex community and ecosystem responses to global environmental change in contrasting environmental settings. Integrating observational, experimental, and statistical techniques may be the most effective strategy for understanding the impact of global change on biological communities and ecosystems. For example, structural equation modeling (SEM) is a powerful multivariate statistical tool (Grace et al., 2012) that enables the testing of the indirect and direct effects of warming and elevation on plant community composition and ecosystem function (Figure 3), explicitly addressing our key questions and hypotheses (see Figure 3). Ongoing climatic change and predictions of average global surface temperatures rising by at least 2°C (and probably considerably more) by the year 2100 (IPCC, 2014) provide an impetus for a better understanding of how long‐term, large‐scale variation in climate influences community and ecosystem processes. SEMs are one potentially useful statistical tool to address questions about how warming may impact biological communities and whole ecosystem functioning. Moving forward, incorporating a wide range of multivariate statistical techniques, such as SEM, linear mixed‐effects models, Bayesian analyses and generalized linear models, with data from manipulative experiments distributed across natural gradients in contrasting environments is a powerful approach to mechanistically understand relationships between communities and ecosystems, and the services derived from ecosystems, undergoing global change.

## 5 MOVING FORWARD

The empirical data generated from the WaRM project can enable a more comprehensive understanding of the impacts of environmental change and species loss on biological communities and ecosystems, and they will help inform and parameterize models about the future of biodiversity, ecosystem functioning, and carbon cycling in contrasting mountain ecosystems around the globe. The fields of biodiversity and ecosystem modeling have developed greatly over the past few decades (Chaplin‐Kramer et al., 2017; Jetz et al., 2019; Thuiller et al., 2019). However, a functional trait‐based approach is needed to bridge the gap between these fields and to simultaneously refine ecosystem models, thereby improving the utility and predictive power of biodiversity forecasts (Funk et al., 2017; van der Plas et al., 2020; Violle et al., 2007). By exploring relationships between species identity and ecosystem functioning, and how the traits of individual organisms may respond to environmental change and in turn affect ecosystems, we may be able to better understand the way ecosystems are structured and predict how they will function in future. Linking experimental manipulations to forecasts of how functional traits impact ecosystem function can be informative, though rarely done, and is a fruitful path forward.

Manipulative experiments are constructed to test mechanisms and thus are often focused on relatively small spatial and temporal scales, limiting the ability to forecast from their data. Now with increased opportunities for international collaboration, it is possible to coordinate networks of manipulative ecological experiments that run simultaneously at macroecological scales (Borer et al., 2014; Fraser et al., 2012; Nogues‐Bravo & Rahbek, 2011). Data from such experiments are more suitable for larger‐scale modeling approaches, especially in community and ecosystem ecology. By combining work along environmental gradients with formal experimental approaches that manipulate both the abiotic environment and interactions among neighbors, we are able to capitalize on the advantages of both approaches (Fukami & Wardle, 2005). Indeed, we suggest that similar distributed network in other systems would be an important step forward for predicting how global change and the reorganization of communities interact to shape ecosystem function.

## AUTHOR CONTRIBUTIONS

**Case M. Prager:** Conceptualization (supporting); data curation (equal); formal analysis (equal); investigation (equal); project administration (supporting); supervision (supporting); writing – original draft (lead); writing – review and editing (lead). **Aimee Classen:** Conceptualization (lead); funding acquisition (lead); investigation (equal); methodology (equal); project administration (equal); resources (equal); supervision (equal); writing – original draft (equal); writing – review and editing (equal). **Maja Sundqvist:** Conceptualization (equal); investigation (equal); methodology (equal); project administration (supporting); resources (supporting); supervision (supporting); writing – original draft (supporting); writing – review and editing (equal). **M Noelia Barrios‐Garcia:** Conceptualization (supporting); investigation (equal); methodology (supporting); project administration (equal); resources (supporting); supervision (equal); writing – review and editing (equal). **Erin Cameron:** Formal analysis (supporting); investigation (supporting); resources (supporting); writing – review and editing (equal). **Litong Chen:** Investigation (supporting); project administration (supporting); resources (supporting); supervision (supporting); writing – review and editing (equal). **Chelsea Chisholm:** Conceptualization (supporting); formal analysis (supporting); investigation (equal); project administration (supporting); resources (supporting); supervision (supporting); writing – review and editing (equal). **Tom Crowther:** Conceptualization (supporting); funding acquisition (supporting); investigation (supporting); methodology (supporting); project administration (supporting); resources (supporting); writing – original draft (supporting); writing – review and editing (equal). **Julie Deslippe:** Conceptualization (equal); funding acquisition (supporting); investigation (equal); methodology (equal); project administration (equal); resources (supporting); supervision (equal); writing – review and editing (equal). **Karl Grigulis:** Investigation (supporting); project administration (supporting); resources (supporting); supervision (supporting); writing – review and editing (supporting). **Jin‐Sheng He:** Conceptualization (equal); formal analysis (equal); funding acquisition (supporting); investigation (lead); methodology (equal); project administration (equal); resources (equal); supervision (equal); writing – review and editing (equal). **Jeremiah Henning:** Data curation (supporting); formal analysis (supporting); project administration (supporting); writing – review and editing (equal). **Mark Hovenden:** Conceptualization (equal); funding acquisition (supporting); investigation (equal); methodology (equal); project administration (equal); resources (supporting); supervision (equal); writing – review and editing (equal). **Toke T. Thomas Hoye:** Conceptualization (equal); funding acquisition (supporting); investigation (equal); methodology (equal); project administration (equal); resources (supporting); supervision (equal); writing – review and editing (equal). **Xin Jing:** Formal analysis (supporting); investigation (supporting); project administration (supporting); writing – review and editing (equal). **Sandra Lavorel:** Investigation (equal); project administration (supporting); resources (supporting); writing – review and editing (equal). **Jennie McLaren:** Conceptualization (equal); funding acquisition (supporting); investigation (equal); methodology (equal); project administration (equal); resources (supporting); supervision (equal); writing – review and editing (equal). **Dan Metcalfe:** Investigation (supporting); project administration (supporting); writing – review and editing (equal). **Greg Newman:** Conceptualization (supporting); data curation (equal); formal analysis (equal); investigation (supporting); methodology (supporting); project administration (supporting); writing – review and editing (equal). **Marie Louise Nielsen:** Data curation (equal); formal analysis (equal); investigation (equal). **Christian Rixen:** Conceptualization (equal); funding acquisition (supporting); investigation (equal); methodology (equal); project administration (equal); resources (supporting); supervision (equal); writing – review and editing (equal). **Quentin Read:** Data curation (supporting); formal analysis (supporting); investigation (supporting); writing – review and editing (equal). **Kenna Rewcastle:** Data curation (supporting); formal analysis (supporting); investigation (supporting); writing – review and editing (equal). **Mariano Rodriguez Cabal:** Conceptualization (equal); funding acquisition (supporting); investigation (equal); methodology (equal); project administration (equal); resources (supporting); supervision (equal); writing – review and editing (equal). **David A. Wardle:** Conceptualization (equal); project administration (supporting); supervision (supporting); writing – review and editing (equal). **Sonia Wipf:** Conceptualization (equal); investigation (equal); methodology (equal); project administration (equal); resources (supporting); supervision (equal); writing – review and editing (equal). **Nate J Sanders:** Conceptualization (lead); data curation (equal); formal analysis (equal); funding acquisition (lead); investigation (equal); methodology (equal); project administration (lead); resources (lead); supervision (lead); writing – original draft (equal); writing – review and editing (equal).

## CONFLICT OF INTEREST

The authors declare that they have no conflict of interest.

## Supporting information


Figure S1

## Data Availability

The data that support this work are presented in Table 1 of this manuscript.
