# Supplementary material for: Integrating natural gradients, experiments, and statistical modeling in a distributed network experiment: An example from the WaRM Network
Source: Ecol Evol. 2022 Oct 17;12(10):e9396. doi: 10.1002/ece3.9396 (PMC9575997; doi:10.1002/ece3.9396)
Supplement: Supplementary file 1 — Figure S1 [file ECE3-12-e9396-s001.docx]

Supplemental Material

Figure S1. Winter precipitation and temperature (defined as the coldest quarter) across the WaRM Network.
